# Feasibility of transitioning from APACHE II to SAPS III as prognostic model in a Brazilian general intensive care unit. A retrospective study: Viabilidade da transição do APACHE II para o SAPS III como modelo prognóstico em uma unidade de terapia intensiva geral no Brasil. Um estudo retrospectivo

**DOI:** 10.1590/1516-3180.2013.8120014

**Published:** 2014-10-17

**Authors:** Ary Serpa, Murillo Santucci Cesar de Assunção, Andréia Pardini, Eliézer Silva

**Affiliations:** I MSc. Physician, Discipline of Critical Care Medicine, Faculdade de Medicina do ABC (FMABC), Santo André, São Paulo, Brazil, Department of Critical Care Medicine, Hospital Israelita Albert Einstein (HIAE), São Paulo, Brazil.; II MD, MSc. Physician, Department of Critical Care Medicine, Hospital Israelita Albert Einstein (HIAE), São Paulo, Brazil.; III BSc. Nurse, Department of Critical Care Medicine, Hospital Israelita Albert Einstein (HIAE), São Paulo, Brazil.; IV MD, PhD. Director of Department of Critical Care Medicine, Hospital Israelita Albert Einstein (HIAE), São Paulo, Brazil.

**Keywords:** APACHE, Mortality, Intensive care units, Prognosis, Intensive care, APACHE, Mortalidade, Unidades de terapia intensiva, Prognóstico, Terapia intensiva

## Abstract

**CONTEXT AND OBJECTIVE:**

Prognostic models reflect the population characteristics of the countries from which they originate. Predictive models should be customized to fit the general population where they will be used. The aim here was to perform external validation on two predictive models and compare their performance in a mixed population of critically ill patients in Brazil.

**DESIGN AND SETTING:**

Retrospective study in a Brazilian general intensive care unit (ICU).

**METHODS:**

This was a retrospective review of all patients admitted to a 41-bed mixed ICU from August 2011 to September 2012. Calibration (assessed using the Hosmer-Lemeshow goodness-of-fit test) and discrimination (assessed using area under the curve) of APACHE II and SAPS III were compared. The standardized mortality ratio (SMR) was calculated by dividing the number of observed deaths by the number of expected deaths.

**RESULTS:**

A total of 3,333 ICU patients were enrolled. The Hosmer-Lemeshow goodness-of-fit test showed good calibration for all models in relation to hospital mortality. For in-hospital mortality there was a worse fit for APACHE II in clinical patients. Discrimination was better for SAPS III for in-ICU and in-hospital mortality (P = 0.042). The SMRs for the whole population were 0.27 (confidence interval [CI]: 0.23 - 0.33) for APACHE II and 0.28 (CI: 0.22 - 0.36) for SAPS III.

**CONCLUSIONS:**

In this group of critically ill patients, SAPS III was a better prognostic score, with higher discrimination and calibration power.

## RESUMO

**CONTEXTO E OBJETIVO:**

Modelos prognósticos refletem as características da população dos países de onde eles são originários. Modelos preditivos devem ser customizados para se adequar à população geral onde eles serão utilizados. O objetivo aqui foi de realizar a validação externa de dois modelos preditivos e comparar o seu desempenho em uma população mista de pacientes graves no Brasil.

**TIPO DE ESTUDO E LOCAL:**

Estudo retrospectivo em uma unidade de terapia intensiva geral brasileira.

**MÉTODOS:**

Este é um estudo retrospectivo de todos os pacientes internados em uma unidade de terapia intensiva (UTI) mista com 41 leitos entre agosto de 2011 e setembro de 2012. A calibração (avaliada com o teste de Hosmer-Lemeshow *goodness-of-fit*) e a discriminação (avaliada como a área sob a curva) do APACHE II e do SAPS III foram comparados. A razão de mortalidade padronizada (SMR) foi calculada pela divisão do número de óbitos observados pelo número de óbitos esperados.

**RESULTADOS:**

Um total de 3.333 pacientes internados na UTI foi registrado. O teste de Hosmer-Lemeshow *goodness-of-fit* demonstrou boa calibração para todos os modelos em relação a mortalidade hospitalar. Para a mortalidade intra-hospitalar, há um ajuste pior do APACHE II em pacientes clínicos. A discriminação foi melhor para o SAPS III para mortalidade na UTI e no hospital (P = 0,042). A SMR para toda a população foi de 0,27 (intervalo de confiança [IC]: 0,23-0,33) para APACHE II e de 0,28 (IC: 0,22-0,36) para SAPS III.

**CONCLUSÕES:**

Neste grupo de pacientes graves, o SAPS III é o melhor escore prognóstico, com a maior discriminação e poder de calibração.

## INTRODUCTION

Prognostic models reflect the population characteristics of the countries from which they originate. The development of the Acute Physiology and Chronic Health Evaluation II (APACHE II) system was based on a cohort of patients in the United States,^1^ and it has been used in many intensive care units around the word. In contrast, the Simplified Acute Physiology Score III (SAPS III) was validated in a multicenter and multinational cohort study.^2^

Predictive models should be customized to fit the case-mix population where they will be used because the outcomes in the original databases and period from which the models were derived may be different from the databases of intensive care units (ICUs) using the models.^3^^,^^4^ It is not clear whether calibration of the established models for local circumstances would enhance their accuracy in stratifying patients.^5^

Although the methods have been reported to adapt well to different periods and case mixes, few studies have formally assessed the models’ predictive accuracy when applied to new populations from other institutions or countries.^6^^,^^7^^,^^8^^,^^9^ In South America, SAPS III was calibrated with a level of 1.3 (i.e. the relationship between observed and predicted mortality was 1.3).^2^^,^^10^ Recently studies have validated SAPS III in different Brazilian cohorts of patients, obtaining good results.^11^^,^^12^^,^^13^

Comparison between observed and predicted mortality rates could serve as an indicator of ICU performance, and lead to overall improvement in healthcare services. However, ICU profiles vary worldwide, depending on the proportions of medical and surgical patients, admission and discharge policies, availability of intermediate care units and staffing with intensive care specialists.^13^

Any transition from a well-established approach to a new one requires caution and validation. Changing APACHE II for SAPS III has some advantages and the most important is the fact that SAPS III is the only prognostic score that included a cohort of patients from South America in its development.

## OBJECTIVE

In the present study, we aimed to perform external validation on two predictive models and directly compare their performance in an independent population of mixed critically ill patients.

## METHODS

### Data collection

This study was approved by the Ethics Committee of Hospital Israelita Albert Einstein and, because of the retrospective nature of the study, the informed consent requirement was waived. The data were collected from all patients admitted to a mixed 41-bed ICU in the tertiary-level private hospital in Brazil from August 2011 to September 2012.

Data were retrospectively collected using APACHE II only between August 2011 and December 2011, and using SAPS III only between May 2012 and September 2012. From January 2012 to April 2012, during a period of calibration, both scores were calculated for all patients admitted to the ICU and were collected for analysis. The data collection practices were standardized and performed by a trained nurse or physician. All data were checked for implausible and outlying values. The data included age, gender and type of admission (clinical, elective surgery or emergency surgery).

### Study population

All ICU admissions were enrolled during the period analyzed. The exclusion criteria were: age < 18 years, missing data and not receiving ICU care. The admissions between January 2012 and April 2012 were used as a validation database to study the performance of APACHE II versus SAPS III for all admissions and in subgroups according to the type of admission.

### Scores and predicted mortalities

The calculations of the individual scores for each model were based on the most disordered physiological values recorded during the first 24 hours of ICU admission for APACHE II and were based on the variables measured one hour before and after ICU admission for SAPS III. The mortality probabilities for APACHE II and SAPS III were calculated using the original equations.^1^^,^^2^

### Performance of the scores

The calibration of the scores was tested using the Hosmer-Lemeshow goodness-of-fit procedure, which was calculated by dividing the admissions into ten deciles according to the risk of death. The chi-square statistics were determined for each decile and summing the chi-square values for the ten deciles resulted in the test value.^14^ A high P value would indicate a good fit for the model. Hosmer-Lemeshow is a test for assessing agreement between the actual and predicted death rates. The discriminative ability of the models was assessed using receiver operating characteristic (ROC) curves and the respective areas under curves (AUC).^15^ The AUC is an expression of the model’s ability to discriminate correctly between survivors and non-survivors.

The standardized mortality ratio (SMR) was calculated using the models by dividing the number of observed deaths by the number of expected deaths. Confidence intervals for the SMR were calculated to test the model’s uniformity-of-fit, using the methods that have been put forward.^16^ The variables were compared between the three periods using analysis of variance (ANOVA). Calibration curves were constructed by plotting the predicted death rates stratified as 5% intervals of mortality risk (*x*-axis) versus observed death rates (*y*-axis). Finally, we constructed a model using Cox regression analysis with APACHE II and SAPS III as independent factors.

Statistical significance was defined as P < 0.05 and the results are presented as mean ± standard deviation unless indicated otherwise. All the statistical procedures were performed using the SPSS 20.0 statistical package (SPSS, Chicago, Illinois, USA).

## RESULTS

### Study population

A total of 3,333 ICU admissions were enrolled until the end of September 2012. The formation of the database is presented in Table 1. The characteristics of the population in the three periods are presented in Table 2. The ICU and hospital mortality and the APACHE II score decreased over time, and the SAPS III score increased during the periods.

**Table 1.**
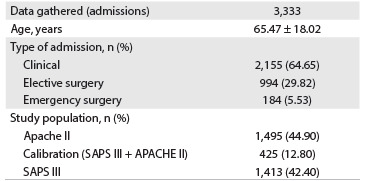
Study database

APACHE II = acute physiology and chronic health disease classification system II; SAPS III = simplified acute physiology score III

**Table 2.**
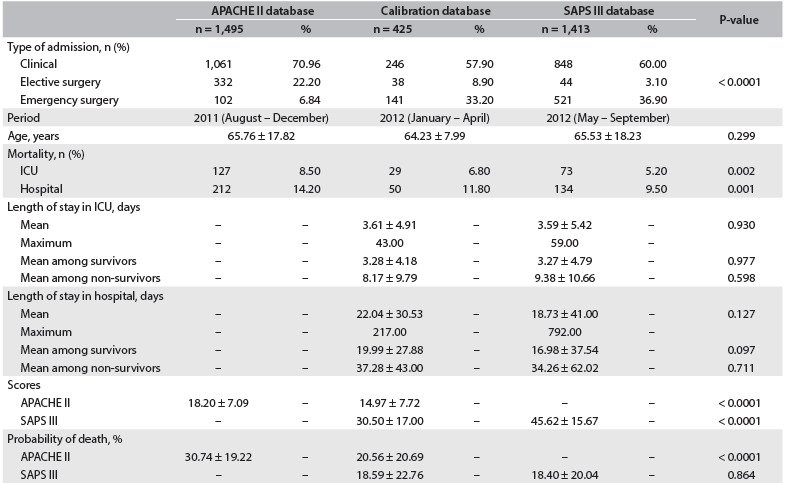
Characteristics of the study population

ICU = intensive care unit; APACHE II = acute physiology and chronic health disease classification system II; SAPS III = simplified acute physiology score III.

### Calibration and discrimination

**Figure 1.**
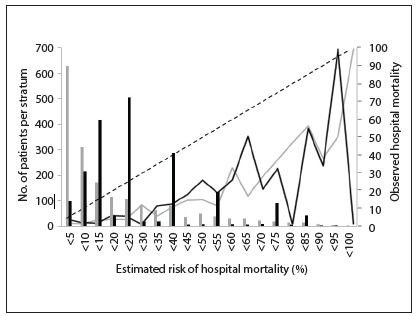
Calibration curve for APACHE II (black line and bar) and SAPS III (gray line and bar*)*. The bars represent the number of patients in each risk group. The dashed diagonal line indicates ideal prediction (predicted = observed mortality).

The Hosmer-Lemeshow goodness-of-fit statistics supported model fit for all in-ICU mortality models with the exception of APACHE II for patients in the calibration database undergoing elective surgery. For in-hospital mortality, there was worse fit for APACHE II among clinical patients during the first period and for SAPS III among patients in the calibration database undergoing elective surgery (Table 3). The calibration curves for APACHE II and SAPS III showed overestimation of the risk of death in all ranges of predicted mortality (Figure 1).

**Table 3.**
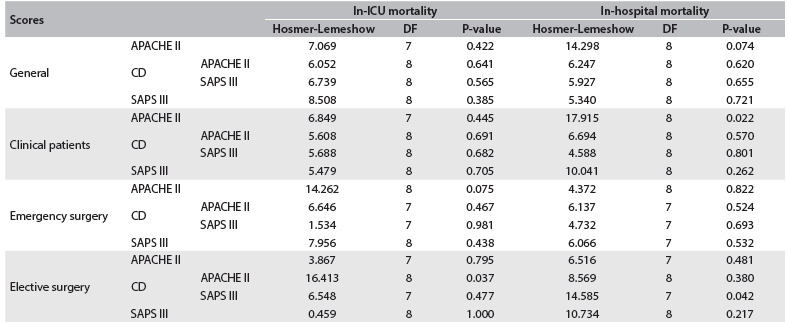
Model calibration assessed by means of Hosmer-Lemeshow goodness-of-fit statistics

ICU = intensive care unit; APACHE II = acute physiology and chronic health disease classification system II; SAPS III = simplified acute physiology score III; DF = degrees of freedom; CD = Calibration database.

**Figure 2.**
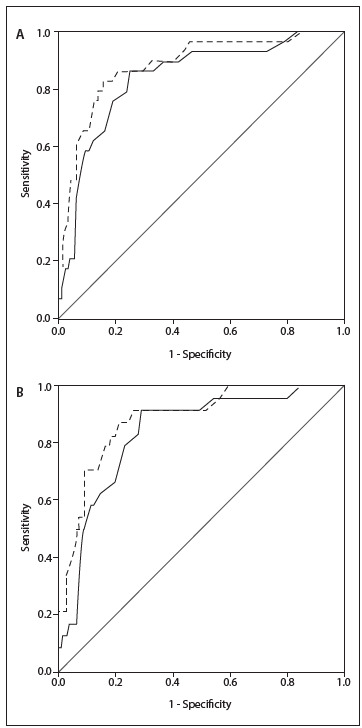
Receiver operating characteristic curve for APACHE II (black line) and SAPS III (black dotted line) in general population (A) and clinical patients (B).

Discrimination, as tested by the AUC, among general and clinical patients, was better for SAPS III in relation to in-ICU and in-hospital mortality (P = 0.042) (Table 4). Figure 2 shows the ROC for SAPS III and APACHE II, for in-ICU mortality in the calibration database in different situations.

**Table 4.**
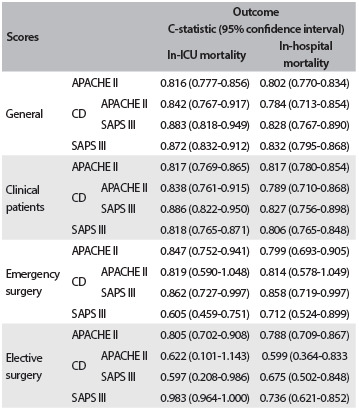
C-statistics (area under receiver operating characteristic curve) as measurement of discrimination between survivors and non-survivors (in-ICU and in-hospital)

ICU = intensive care unit; APACHE II = acute physiology and chronic health disease classification system II; SAPS III = simplified acute physiology score III; CD = Calibration database.

### Standardized mortality ratio

The SMRs for the whole population were 0.27 (CI: 0.23 - 0.33) for APACHE II and 0.28 (CI: 0.22 - 0.36) for SAPS III. In the calibration database, the SMRs for APACHE II and SAPS III were 0.33 (CI: 0.22 - 0.50), and 0.36 (CI: 0.25 - 0.55), respectively. For all models, the SMRs showed some variation across the spectrum of patients. The SMRs ranged from 0.24 to 0.46 for APACHE II, and from 0.09 to 0.31 for SAPS III. In the calibration database, the SMRs ranged from 0.13 to 0.38 for APACHE II, and from 0.18 to 0.40 for SAPS III (Table 5).

**Table 5.**
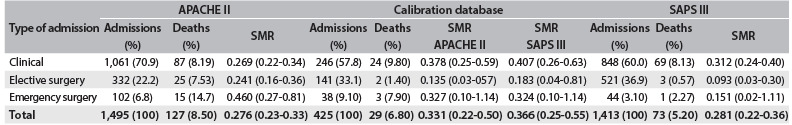
Standardized mortality ratio calculated using the prediction models according to type of admission (95% confidence interval)

ICU = intensive care unit; APACHE II = acute physiology and chronic health disease classification system II; SAPS III = simplified acute physiology score III; SMR: standardized mortality ratio.

### Cox regression model

The hazard ratios for in-hospital and in-ICU mortality using APACHE II as an independent factor were 1.08 (95% CI: 1.04 - 1.12) and 1.09 (95% CI: 1.04 -1.14), respectively. For SAPS III, the hazard ratios for in-hospital and in-ICU mortality were 1.03 (95% CI: 1.03 - 1.04) and 1.04 (95% CI: 1.03 - 1.06), respectively.

## DISCUSSION

The external validation of these two widely used prognostic models showed good discrimination and good calibration when applied to the same independent population of Brazilian ICU patients. The transition from APACHE II to SAPS III in this Brazilian ICU was feasible and, in some scenarios, SAPS III had even better performance than APACHE II.

The SAPS III score was developed using data from 16,784 patients.^2^ However, it was not developed to be representative of all ICU patients, since it was developed in a cohort of general patients. Therefore, external validation is extremely important before applying this score to any type of patient. The cohort used for the conception of the SAPS III model involved patients from Brazilian ICUs, which might explain its superiority to APACHE II in our study. Also, the SAPS III model is based exclusively on data evaluated during the first hour of admission to the ICU.^2^ Prognostic systems that include measurements during and/or after the first 24 hours of admission, like APACHE II, often reflect standard care and not the real clinical status of the patient.

Severity scores have the aim of measuring the severity of the disease of ICU patients and are proposed as tools to aid in outcome assessment and resource allocation. Furthermore, they are used for comparisons of outcomes and quality of care between ICUs.^5^

Our findings are supported by other studies published in the literature. Soares et al.^11^ demonstrated that SAPS III had excellent discrimination in Brazilian ICUs and that APACHE II was unsatisfactory due to its lower discriminatory power and lack of calibration for some populations. However, they also found that SAPS III overestimated hospital mortality, as also found in our study. In a cohort of Brazilian patients, Silva Júnior et al.^17^ concluded that SAPS III is a useful tool for determining which patients will need more care, and for the evolution of high-risk surgical patients.

Alves et al.^18^ showed, among elderly patients admitted to an ICU, that SAPS III had excellent discrimination, but that the calibration was inadequate. Soares et al.^12^ also showed that SAPS III had good discrimination and inadequate calibration among cancer patients. Costa e Silva et al.^19^ showed, in Brazilian critically ill patients with acute kidney injury, that SAPS III presented good discrimination and calibration performances, accurately predicting mortality in this group of patients. Finally, Nassar et al.^13^ demonstrated that SAPS III had good discrimination and inadequate calibration in a general cohort of Brazilian patients.

Our findings are supported by several studies that compared APACHE II with SAPS in different scenarios.^20^^,^^21^^,^^22^ These scores have also already been used with other aims like estimation of prolonged mechanical ventilation in surgical patients.^23^ In our hospital, we have a high number of liver transplantation procedures, and the performance of these scores in these population is a matter of debate.^24^^,^^25^^,^^26^ Also, one limitation of this study is that our hospital has a lower mortality rate than other institutions from Brazil and this makes it difficult to differentiate whether we have a score with bad performance or whether we have an ICU with excellent performance.

## CONCLUSIONS

We showed, in a Brazilian cohort of critically ill patients, that SAPS III was a better prognostic score, with higher discrimination and calibration power. The transition from APACHE II to SAPS III was feasible in this scenario.

## References

[1] Knaus WA, Draper EA, Wagner DP, Zimmerman JE (1985). APACHE II: a severity of disease classification system. Crit Care Med.

[2] Metnitz PG, Moreno RP, Almeida E (2005). SAPS 3--From evaluation of the patient to evaluation of the intensive care unit. Part 1: Objectives, methods and cohort description. Intensive Care Med.

[3] Metnitz PG, Valentin A, Vesely H (1999). Prognostic performance and customization of the SAPS II: results of a multicenter Austrian study. Simplified Acute Physiology Score. Intensive Care Med.

[4] Moreno R, Morais P (1997). Outcome prediction in intensive care: results of a prospective, multicentre, Portuguese study. Intensive Care Med.

[5] Suistomaa M, Niskanen M, Kari A, Hynynen M, Takala J (2002). Customized prediction models based on APACHE II and SAPS II scores in patients with prolonged length of stay in the ICU. Intensive Care Med.

[6] Beck DH, Smith GB, Pappachan JV, Millar B (2003). External validation of the SAPS II, APACHE II and APACHE III prognostic models in South England: a multicentre study. Intensive Care Med.

[7] Wong DT, Crofts SL, Gomez M, McGuire GP, Byrick RJ (1995). Evaluation of predictive ability of APACHE II system and hospital outcome in Canadian intensive care unit patients. Crit Care Med.

[8] Markgraf R, Deutschinoff G, Pientka L, Scholten T (2000). Comparison of acute physiology and chronic health evaluations II and III and simplified acute physiology score II: a prospective cohort study evaluating these methods to predict outcome in a German interdisciplinary intensive care unit. Crit Care Med.

[9] Moreno R, Miranda DR, Fidler V, Van Schilfgaarde R (1998). Evaluation of two outcome prediction models on an independent database. Crit Care Med.

[10] Moreno RP, Metnitz PG, Almeida E (2005). SAPS 3--From evaluation of the patient to evaluation of the intensive care unit. Part 2: Development of a prognostic model for hospital mortality at ICU admission. Intensive Care Med.

[11] Soares M, Salluh JI (2006). Validation of the SAPS 3 admission prognostic model in patients with cancer in need of intensive care. Intensive Care Med.

[12] Soares M, Silva UV, Teles JM (2010). Validation of four prognostic scores in patients with cancer admitted to Brazilian intensive care units: results from a prospective multicenter study. Intensive Care Med.

[13] Nassar AP, Mocelin AO, Nunes AL (2012). Caution when using prognostic models: a prospective comparison of 3 recent prognostic models. J Crit Care.

[14] Hosmer DW, Lemeshow S (1989). Applied logistic regression.

[15] Hanley JA, McNeil BJ (1982). The meaning and use of the area under the receiver operating characteristic (ROC) curve. Radiology.

[16] Gardner MJ, Altman DG (1986). Confidence intervals rather than P values: estimation rather than hypothesis testing. BMJ.

[17] Silva JM, Malbouisson LM, Nuevo HL (2010). Applicability of the simplified acute physiology score (SAPS 3) in Brazilian hospitals. Rev Bras Anestesiol.

[18] Alves CJ, Franco GPP, Nakata CT (2009). Avaliação de índices prognósticos para pacientes idosos admitidos em unidades de terapia intensiva [Evaluation of prognostic indicators for elderly patients admitted in intensive care units]. Rev Bras Ter Intensiva.

[19] Costa e Silva VT, de Castro I, Liaño F (2011). Performance of the third-generation models of severity scoring systems (APACHE IV, SAPS 3 and MPM-III) in acute kidney injury critically ill patients. Nephrol Dial Transplant.

[20] Khwannimit B, Bhurayanontachai R (2009). The performance of customised APACHE II and SAPS II in predicting mortality of mixed critically ill patients in a Thai medical intensive care unit. Anaesth Intensive Care.

[21] Beck DH, Smith GB, Taylor BL (2002). The impact of low-risk intensive care unit admissions on mortality probabilities by SAPS II, APACHE II and APACHE III. Anaesthesia.

[22] McNelis J, Marini C, Kalimi R (2001). A comparison of predictive outcomes of APACHE II and SAPS II in a surgical intensive care unit. Am J Med Qual.

[23] Kern H, Redlich U, Hotz H (2001). Risk factors for prolonged ventilation after cardiac surgery using APACHE II, SAPS II, and TISS: comparison of three different models. Intensive Care Med.

[24] Chung SW, Kirkpatrick AW, Kim HL, Scudamore CH, Yoshida EM (2000). Correlation between physiological assessment and outcome after liver transplantation. Am J Surg.

[25] Umbro I, Tinti F, Mordenti M (2011). Model for end-stage liver disease score versus simplified acute physiology score criteria in acute renal failure after liver transplantation. Transplant Proc.

[26] Bein T, Fröhlich D, Pömsl J, Forst H, Pratschke E (1995). The predictive value of four scoring systems in liver transplant recipients. Intensive Care Med.

